# A Psychometric Comparison of Faculty-Authored and Large Language Model-Generated Multiple-Choice Questions in Endodontics

**DOI:** 10.7759/cureus.114830

**Published:** 2026-08-20

**Authors:** Pradipkumar Damor, Sarita Gill, Wasim Wani, Gauri Arora, Mayank Charan

**Affiliations:** 1 Department of Conservative Dentistry and Endodontics, School of Dental Sciences, Manav Rachna Dental College, Manav Rachna International Institute of Research and Studies, Faridabad, IND; 2 Department of Conservative Dentistry and Endodontics, Eklavya Dental College and Hospital, Kotputli, IND; 3 Division of Conservative Dentistry and Endodontics, Centre for Dental Education and Research, All India Institute of Medical Sciences, New Delhi, IND; 4 Division of Conservative Dentistry and Endodontics, Army Dental Centre (Research and Referral), New Delhi, IND

**Keywords:** artificial intelligence in education, artificial intelligence in endodontics, bloom's taxonomy, chatbots, dental education, educational assessment, item difficulty, question bank development, reliability analysis, test validity

## Abstract

Background and objective

Designing high-quality multiple-choice questions (MCQs) in dental education is highly resource-intensive. While generative artificial intelligence (AI) offers a rapid, scalable solution for automated item generation, there is limited student-tested psychometric evidence comparing uncurated large language model (LLM) outputs with traditional faculty-authored items in advanced dental specialties like endodontics. This study aimed to evaluate and compare the psychometric properties, specifically the difficulty index, discrimination index, distractor effectiveness, and internal consistency reliability, of faculty-authored and uncurated, LLM-generated endodontic MCQs.

Materials and methods

Sixty single-best-answer MCQs (30 authored by experienced endodontic faculty and 30 generated by the LLM Claude Sonnet 5 (Anthropic, San Francisco, CA) using a standardized single-prompt framework) were distributed evenly across 10 core endodontic domains. The unified 60-item examination was digitally administered to a convenience sample of 100 dental interns in a single, randomized, 60-minute session. Psychometric parameters were computed using classical test theory, and scale reliabilities were evaluated using Cronbach’s alpha (*α*). Continuous variables were compared using independent-samples t-tests.

Results

The faculty-authored subscale demonstrated good internal consistency (*α* = 0.851), while the LLM subscale showed acceptable reliability (*α* = 0.798). Faculty-authored items had a significantly higher mean discrimination capacity (*p* = 0.00747) and a more balanced difficulty profile, with 27 (90.0%) of the items falling into the moderate difficulty range. Conversely, LLM-generated items were significantly easier (*p* = 0.000918), with 13 (43.3%) classified as easy, and had a high rate of non-functional distractors (NFDs). While five (16.7%) of the LLM questions had 100% NFDs (where all distractors failed to function), the faculty cohort had no such items. A concurrency analysis revealed that three (10.0%) of the faculty items simultaneously satisfied all ideal psychometric benchmarks, compared to only one (3.3%) of the LLM items.

Conclusions

Although next-generation LLMs can produce stylistically authentic clinical vignettes that closely mimic human-authored writing, their raw, uncurated outputs exhibit significant psychometric limitations, particularly regarding distractor plausibility and item discrimination. Fully autonomous AI item generation is not yet viable for high-stakes assessments. A hybrid “human-in-the-loop” model, where LLMs are leveraged for rapid preliminary drafting and experienced educators perform targeted distractor refinement, can safeguard assessment validity while substantially reducing the administrative burden on dental faculty.

## Introduction

Multiple-choice questions (MCQs), especially single-best-answer clinical vignettes, are the dominant format for objective knowledge assessment in health professions education because they enable standardized, reliable testing across large cohorts and can assess applied clinical reasoning when well-constructed [1]. In dental education, MCQs are considered a cornerstone of assessment, with their value tied to fairness, objectivity, curriculum coverage, and post-test psychometric review that sustains validity and reliability over time [2,3]. This is particularly relevant in clinically oriented dental disciplines, where assessments must distinguish simple recall from interpretation and application. Postgraduate and advanced dental undergraduate education has therefore focused specifically on single-correct-option items, cognitive level, item-writing flaws, and difficulty characteristics, underscoring the centrality of rigorous item design to the quality of specialty training [2,4].

The problem is that writing such items well is difficult. Recent medical and dental education studies consistently describe high-quality question development as laborious, time-consuming, and resource-intensive work requiring both domain expertise and methodological skill in blueprinting, distractor construction, quality assurance, and post-examination analysis [3,5,6]. Faculty must align items with learning outcomes, avoid technical flaws, calibrate cognitive level, and revise or discard weak questions after psychometric testing, all of which amplify faculty workload and contribute to assessment fatigue, especially in content-dense clinical curricula, and often require repeated faculty development to improve performance [2-5].

Generative artificial intelligence (AI) is therefore an attractive solution for automated item generation. Across recent studies, large language models (LLMs) are presented as a scalable means of producing questions rapidly, with substantial efficiency gains compared with conventional authorship. Empirical estimates of this advantage are large: models have reduced item generation time compared with manual human authorship [1,6-8]. Thus, frontier models can help expand item banks and improve operational feasibility, particularly where institutions face increasing demands for frequent, blueprint-aligned assessment [3,9].

Yet recent literature also reveals a psychometric paradox: raw outputs frequently underperform on the psychometric properties that determine assessment quality [10]. Dental and medical comparisons show that faculty-authored items often retain stronger discrimination, broader difficulty distributions, and better alignment of distractors with student misconceptions, whereas raw LLM outputs often require exclusion or revision because of repetition or bias [3,5]. Distractor mechanics appear especially decisive; expert evaluations identify distractor uniqueness as challenging for LLMs, and studies of generated items repeatedly note the need for expert review to correct weak distractors and preserve discrimination [1,10].

Van der Vleuten’s utility framework provides the appropriate conceptual lens for interpreting this trade-off; trustworthy exam design is a multiplicative function of validity, reliability, educational impact, acceptability, and cost-efficiency or feasibility [8,11]. Within that model, feasibility gains from rapid automated generation cannot compensate for a total failure in validity or reliability. This concern is acute in dental education, where limited evidence exists on AI use beyond board-style items, and where unchecked model outputs can undermine high-quality discrimination at the critical boundary between acceptable and unacceptable clinical competence [12].

A critical gap therefore remains. Despite growing comparisons of AI- and human-authored questions in dental specialties, raw, student-tested psychometric validation focused on highly constrained, prompt-governed frontier-model item generation in an advanced dental subspecialty such as endodontics is lacking. Existing positive findings are also often contingent on expert filtering, dual-model validation, fine-tuning, or other human-in-the-loop safeguards, which confound direct inference about the psychometric behavior of generated items themselves. Accordingly, the objective of the present study was to evaluate and compare the student-tested item difficulty index, discrimination index, distractor efficiency, and internal consistency reliability of faculty-authored and LLM-generated MCQs in endodontics among dental interns.

## Materials and methods

Study setting and ethical considerations

**Figure 1:**
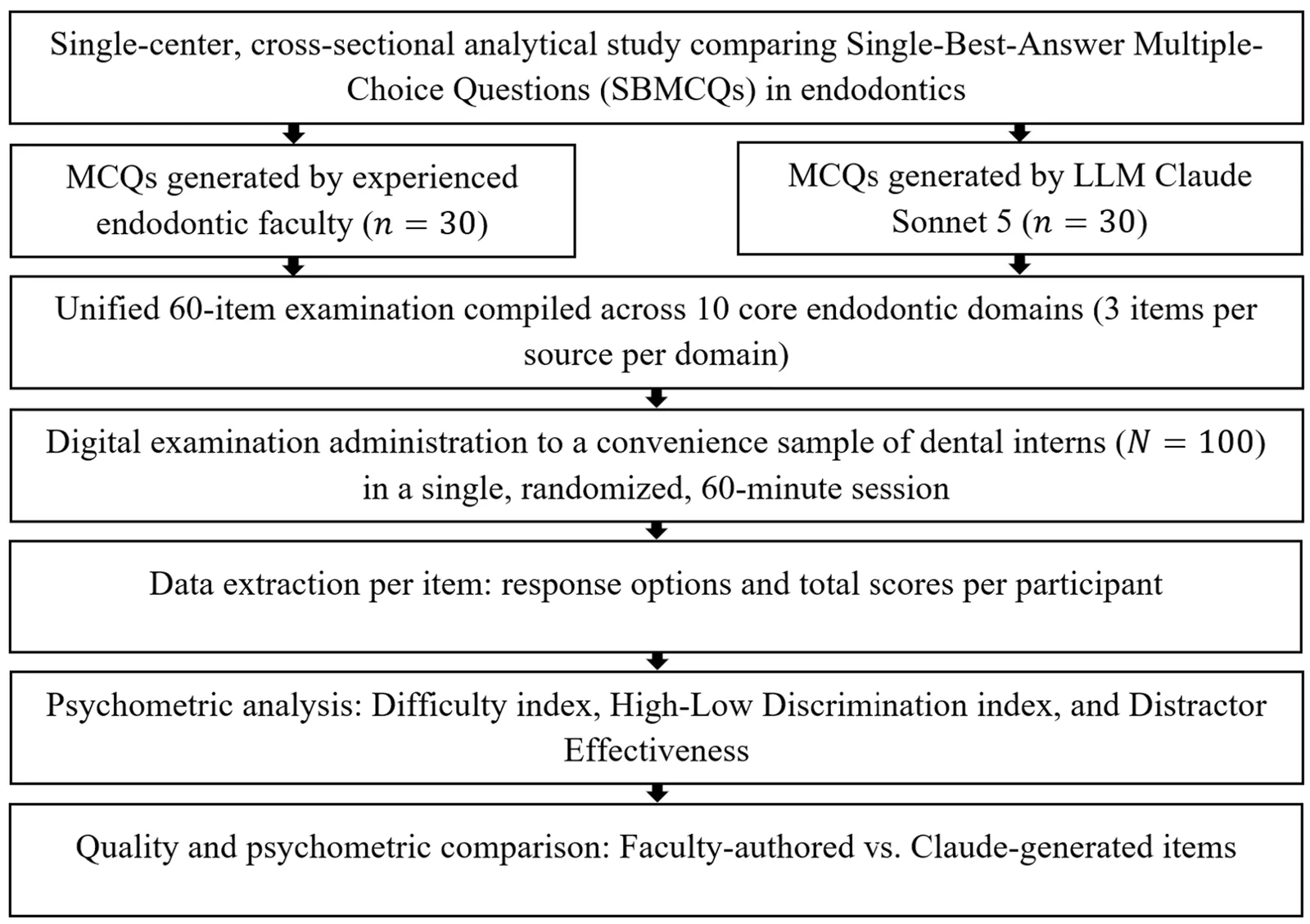
Methodological flowchart of the study design The diagram illustrates the comparative workflow of item generation, test composition across 10 core endodontic domains, administration to dental interns (N = 100), and subsequent psychometric analysis evaluating item difficulty, high-low discrimination, and distractor effectiveness MCQs: multiple-choice questions; LLM: large language model

This single-center, comparative psychometric evaluation was conducted at the Department of Conservative Dentistry and Endodontics, Manav Rachna Dental College, among Bachelor of Dental Surgery students enrolled in the compulsory rotatory internship. The study protocol was approved by the Institutional Ethics Committee (IEC) (Approval No: MRDC/IEC/2025/100) before implementation. Participation was voluntary, and all student response data were anonymized before statistical analysis to ensure data integrity and participant confidentiality. A comprehensive visual overview of the sequential workflow, including item generation, digital test administration, and psychometric evaluation, is illustrated in Figure 1.

Curricular blueprinting and domain selection

Ten core endodontic domains were selected based on the official undergraduate BDS curriculum guidelines to ensure focus on foundational and essential clinical topics. To eliminate topic bias and ensure a balanced curricular distribution, a total test bank of 60 single-best-answer MCQs was established, allocating six items per domain. The foundational clinical content for all items was sourced from standard reference textbooks: Cohen’s Pathways of the Pulp, twelfth edition, and Ingle’s Endodontics, seventh edition.

Item generation

Faculty-Authored MCQs

Two experienced endodontic faculty members, each possessing more than four years of teaching experience and having undergone standardized institutional training in item-writing principles, generated a total of 30 MCQs distributed evenly across the ten domains (three items per domain). To ensure true structural equity and control for baseline writing quality, the faculty authors were required to follow the structural guidelines embedded within the prompt framework.

LLM-Generated MCQs

Item generation was executed utilizing the LLM Claude Sonnet 5 (Anthropic, San Francisco, CA) in July 2026. Interaction with the model was governed via a single-prompt framework developed with support from a prompt-engineering expert. In accordance with the model’s application programming interface (API) architecture, custom sampling parameters (temperature, top-p, top-k) are non-configurable and rejected by default, with generation governed via adaptive thinking operating under native platform effort settings. To maximize reproducibility, default web generation settings were maintained. One experienced endodontic investigator manually selected and inputted each target endodontic domain into the prompt framework. The prompt enforced strict rules regarding option homogeneity, distractor plausibility, and formatting style, and required the LLM to output the active domain, intended question style, and targeted cognitive level.

To balance domain consistency with item independence, a fresh chat session was initialized for each endodontic domain. This enabled intra-domain context awareness to prevent duplicate questions while ensuring cross-domain isolation. The master prompt framework (detailed in Appendix 1), specific domain titles, and their operational definitions (detailed in Appendix 2) were fed verbatim into the model. A total of 30 LLM-drafted MCQs were generated. A representative example of the raw, unedited model output is provided in Appendix 3. No outputs were discarded or re-prompted; all generated items were integrated into the test bank in their native state for expert audit, thereby eliminating selection bias and ensuring full transparency of the baseline model performance.

Content validation and blinding

Before test administration, the combined pool of 60 randomized and coded items was audited by an independent panel of two external endodontic experts who were completely blinded to the source of the items and had no involvement in the original generation of the MCQs. The panel reviewed the items solely to verify clinical accuracy, safety, and blueprint alignment. All 60 generated items (30 faculty-authored and 30 LLM-generated) met all expert audit criteria for clinical accuracy, safety, and blueprint alignment on initial review without requiring exclusion or structural replacement. Additionally, the items were qualitatively evaluated by ten independent endodontic reviewers to determine the perceived item source (faculty-authored versus LLM-drafted). Inter-rater agreement for this source-classification task was evaluated using Cohen's Kappa (κ).

Participants and test administration

A convenience sampling approach was utilized, involving the entire eligible cohort of dental interns (*N* = 100). Inclusion criteria required active enrollment in the internship program and attendance on the scheduled examination day. Exclusion criteria included incomplete examination submissions, absence on the testing day, or unwillingness to participate. To eliminate practice effects, memory/exposure biases, and context effects associated with multi-session designs, the assessment was deployed as a single, unified, 60-item digital examination using Google Forms (Google LLC, Mountain View, CA). To eliminate source clustering, order effects, and sequence bias, the "shuffle question order" feature was enabled, generating a unique, randomized item sequence for each participant. The exam was completed in a single, supervised one-hour session. No negative marking was applied, and students were completely blinded to the source of the items. Proctors and data analysts remained fully blinded until final data lock.

Psychometric and statistical analysis

Student responses were exported and compiled into a master spreadsheet using Microsoft Excel 365 (Microsoft Corporation, Redmond, WA) and coded dichotomously, where a score of one indicated a correct answer and zero indicated an incorrect answer. Every item was comprehensively evaluated across three core psychometric indices.

Difficulty Index

The difficulty index (*P*) was calculated as the overall proportion of examinees answering the item correctly using the formula: *P = R / N*, where R represents the number of correct responses and N represents the total number of examinees. Items were stratified into three distinct performance tiers: easy (*P* > 0.85), moderate (0.30 ≤ *P* ≤ 0.85), and hard (*P* < 0.30).

Item Discrimination Index (D) and Extreme-Groups Analysis

To optimize the statistical power and stability of the discrimination indices, the student population was ordered by cumulative test performance. The upper and lower extreme performance groups were defined using Kelley’s 27% rule. Students falling within the middle 46% of the distribution were excluded solely from the calculation of the discrimination index, though their scores contributed to the overall cohort metrics. The discrimination index for each item was computed using the formula: *D = (U - L) / n*, where *U* is the number of correct responses in the upper extreme group (*n* = 27), *L* is the number of correct responses in the lower extreme group (*n* = 27), and *n* represents the total number of students within a single extreme group (*n *= 27). The values were interpreted as follows: values greater than or equal to 0.40 indicated excellent discrimination; 0.30 to 0.39 indicated good; 0.20 to 0.29 indicated marginal or fair; 0.00 to 0.19 indicated poor or low.

Distractor Effectiveness

Distractor effectiveness was determined by tracking the selection patterns of options. A non-functioning distractor (NFD) was defined as any distractor selected by less than 5% of the total examinees, corresponding to four or fewer students. Item distractor effectiveness was scored continuously based on functioning distractors, where zero NFDs represented a 100% excellent rating, one NFD represented a 66.6% good rating, two NFDs represented a 33.3% moderate rating, and three NFDs represented a 0% poor rating.

Scale Internal Consistency and Reliability

Scale internal consistency and reliability were calculated independently for the 30-item faculty subscale and the 30-item LLM subscale using Cronbach's alpha (*α*). Reliability ranges were interpreted as follows: values greater than or equal to 0.90 were excellent, 0.80 to 0.89 were good, 0.70 to 0.79 were acceptable, 0.60 to 0.69 were questionable, and values less than 0.60 were poor.

Data normality was formally evaluated using the Shapiro-Wilk test. Continuous psychometric parameters meeting normality assumptions (*p* > 0.05) were analyzed using Student’s independent-samples t-test. For parameters exhibiting a departure from normal distribution (*p* < 0.05), non-parametric comparison was conducted using the Mann-Whitney U test. Where parametric testing was applicable, effect sizes were calculated using Cohen's *d* and interpreted as small (*d* = 0.2), medium (*d* = 0.5), or large (*d* = 0.8). Item discrimination index and distractor effectiveness were designated as primary psychometric outcomes, whereas source-classification accuracy and concurrency rates were analyzed as secondary/exploratory outcomes. Significance was established at *p* < 0.05 across all tests. All statistical calculations were performed in Microsoft Excel 365.

## Results

**Figure 2:**
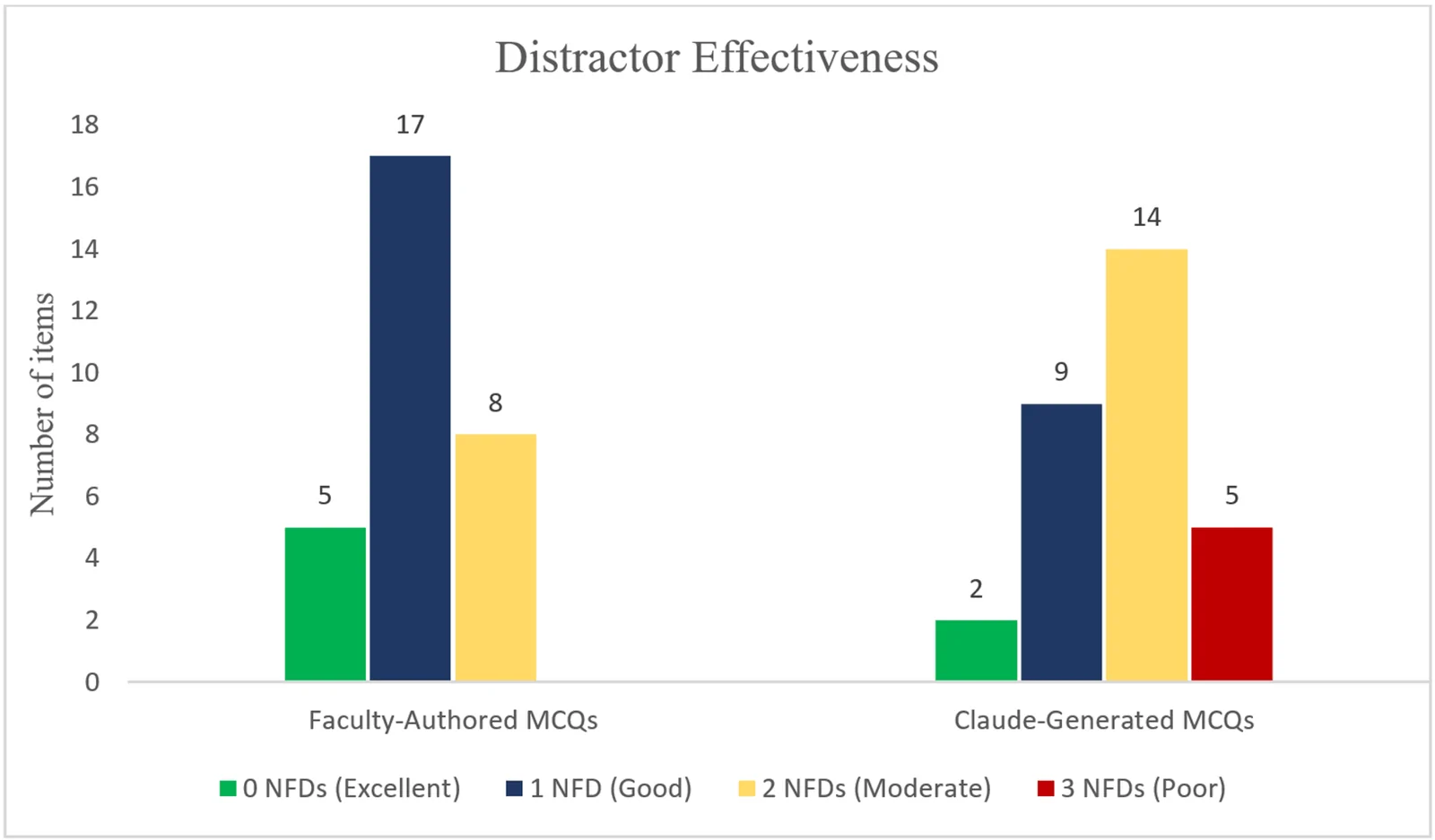
Comparative distribution of distractor effectiveness between faculty-authored and Claude-generated MCQs The bar chart illustrates the frequency distribution of items categorized by the number of NFDs across both groups (n = 30 items per group). Item quality classifications are defined as: 0 NFDs (excellent), 1 NFD (good), 2 NFDs (moderate), and 3 NFDs (poor) MCQs: multiple-choice questions; NFDs: non-functioning distractors

The 30-item faculty-authored subscale demonstrated good internal consistency (*α* = 0.851), while the LLM-generated subscale exhibited acceptable reliability (*α* = 0.798). Inferential evaluation revealed significant differences in continuous psychometric performance between the two cohorts. A Mann-Whitney U test indicated that LLM-generated items were significantly easier than faculty-authored items (LLM median = 0.840 vs. faculty median = 0.715; U = 220.5, *p* < 0.001). Conversely, independent-samples t-tests showed that faculty-authored items demonstrated significantly higher baseline discrimination capacity: *p* = 0.007, mean difference = 0.16, 95% CI (0.04, 0.27), Cohen's *d* = 0.72. The categorical distribution of item difficulty and discrimination tiers is summarized in Table 1. Structural analysis of the 90 wrong-answer alternatives per cohort showed that faculty items possessed superior distractor utility compared to LLM-generated items (Figure 2). While the faculty-authored cohort had zero items classified as pedagogically "poor" (three NFDs), the uncurated LLM cohort contained five items (16.7%) where every single distractor failed to function.

**Table 1:** Psychometric stratification of difficulty and discrimination indices

Index and performance tier	Faculty items (n = 30)	Claude items (n = 30)
Difficulty index		
Easy (p > 0.85)	3 (10.0%)	13 (43.3%)
Moderate (p = 0.30 to 0.85)	27 (90.0%)	17 (56.7%)
Hard (p < 0.30)	0 (0.0%)	0 (0.0%)
Discrimination index		
Excellent (D ≥ 0.40)	19 (63.3%)	10 (33.3%)
Good (D = 0.30 to 0.39)	5 (16.7%)	2 (6.7%)
Marginal/fair (D = 0.20 to 0.29)	4 (13.3%)	7 (23.3%)
Poor/low (D = 0.00 to 0.19)	2 (6.7%)	11 (36.7%)

In the source-classification task, inter-rater agreement for classifying items as faculty-authored or LLM-drafted across the reviewers was *κ* = 0.152, with an observed agreement (*P_o_*) of 0.584 and an expected agreement (*P_e_*) of 0.510, indicating slight agreement beyond chance. Reviewer-level accuracies ranged from 10% to 40% (mean ≈ 29%), consistent with slight agreement.

To evaluate the holistic pedagogical utility of both item generation methods, a concurrency analysis was executed. This isolated the specific subset of questions within each cohort that simultaneously fulfilled all three optimal psychometric benchmarks: an appropriate difficulty index (*p* = 0.30 to 0.85), excellent or good discriminatory power (*D* ≥ 0.30), and total distractor optimality (zero non-functional distractors, representing 100% distractor effectiveness). Within the faculty-authored cohort, three items (10.0%) successfully satisfied all three ideal psychometric criteria simultaneously. In contrast, the LLM-generated cohort yielded one item (3.3%) that concurrently satisfied all three parameters.

## Discussion

This study evaluated the psychometric viability and structural characteristics of MCQs in endodontics generated by the frontier LLM Claude Sonnet 5 compared to those authored by experienced academic faculty. Under the guidance of classical test theory and Van der Vleuten’s utility framework, the findings offer a nuanced perspective on the capabilities and limitations of generative AI in clinical assessment design. By utilizing a prompt framework that granted the model full autonomy over item formatting, the LLM natively generated clinical vignettes at the cognitive level of apply/analyze for 23 items (76.7%) of its items. This spontaneous formatting preference suggests that the model's underlying training architecture recognizes endodontic competencies as complex, multi-variable clinical problems that are best assessed through higher-order cognitive processing rather than simple factual recall.

One of the interesting findings of this study was the outcome of the expert source-classification task, where seasoned academic evaluators were effectively unable to distinguish between faculty-authored and LLM-generated items. The resulting inter-rater agreement barely exceeded random chance, while the mean reviewer-level classification accuracy fell substantially below the 50% probability of random guessing, demonstrating that Claude Sonnet 5 achieved stylistic mimicry. By successfully adopting the formal prose, precise nomenclature, and structural formatting characteristic of professional dental education literature, the model produced no awkward syntax, robotic phrasing, or structural markers that would flag it as synthetic. Because the prompt framework enforced strict rules on option homogeneity and clinical formatting, the items bypassed the typical qualitative detection mechanisms of academic reviewers. From a validation perspective, within the scope of this study, the LLM successfully cleared the hurdle of writing authenticity, achieving 100% baseline clinical and technical accuracy during pre-test expert auditing with zero factually incorrect keys or unsafe advice.

Indeed, the objective psychometric data paint a completely different story, illustrating that the linguistic appearance of quality did not translate to pedagogical utility. The statistical analysis revealed that the LLM-generated items were significantly easier than the faculty-authored items (*p* < 0.001). While the faculty successfully anchored 27 items (90.0%) of their questions within the optimal moderate difficulty range (0.30 to 0.85), nearly half of the LLM-generated items, 13 items (43.3%), fell into the easy category, compared to just three items (10.0%) of the faculty items. This difficulty gap likely stems from the fundamental nature of information retrieval in generative AI. These models excel at compiling, rephrasing, and outputting highly structured factual information directly from reference materials, which frequently results in explicit, linear linkages between the clinical stem and the correct answer [13]. In contrast, experienced human educators intuitively weave clinical nuances, varied diagnostic terminology, and multi-step diagnostic reasoning into their items, naturally elevating the cognitive load and creating a more balanced difficulty distribution.

This structural discrepancy directly impacted the item discrimination index, where the faculty outperformed the LLM cohort to a highly significant degree (*p* = 0.007). Faculty-authored questions achieved excellent discrimination on 19 items (63.3%), clearly separating high-performing students from their lower-performing peers. Conversely, LLM items exhibited a highly dispersed profile, with 11 items (36.7%) flagged as psychometrically poor or low in discrimination. While faculty possess an implicit, localized understanding of student misconceptions, common diagnostic pitfalls in the clinic, and curriculum weaknesses, the uncurated model lacks this contextual calibration. Operating without real-world feedback or historical student performance data, its questions frequently become either too straightforward or lack the precise clinical positioning required to separate top-tier students from lower-performing ones.

In classical test theory, the quality of an MCQ relies heavily on functional distractors to actively engage lower-performing students [14]. In this study, the faculty-authored MCQs demonstrated superior distractor utility, with no items classified as psychometrically poor and over 22 items (73.3%) falling into the excellent or good tiers. Conversely, five items (16.7%) of the LLM-generated questions were categorized as poor, driven by a high prevalence of non-functioning distractors (NFDs). While the model stringently adhered to the prompt instructions regarding option homogeneity and grammatical consistency, it frequently generated distractors that were technically accurate but clinically superficial to an advanced dental intern. This shows that the model can easily enforce the syntax and structure of a professional exam item, but it struggles to replicate the substance of authentic clinical distraction.

This deficit in distractor effectiveness was a primary driver behind the model's lower discrimination indices and elevated ease. Beyond the items with three NFDs, 14 (46.7%) model-generated items suffered from two NFDs. When an MCQ contains two or three NFDs, it effectively ceases to operate as a four-option item. Students can easily eliminate the superficial options, transforming a complex clinical vignette into a basic binary choice or a direct recall task. This structural distractor collapse artificially inflates the difficulty index by making the item easier and severely compresses the discrimination index, as weaker students can easily guess the correct answer. These findings demonstrate that while the model can draft credible distractors grammatically, it struggles to make them cognitively challenging to actual students in a live testing environment.

The true extent of this pedagogical gap is highlighted when evaluated through a multidimensional concurrency analysis. Designing MCQs is a demanding academic task that requires an item to simultaneously satisfy three strict, independent psychometric benchmarks: a moderate difficulty index, excellent or good discriminatory power, and total distractor optimality (zero NFDs). When this strict concurrent filter was applied, the pool of viable questions shrank dramatically in both cohorts, illustrating the immense difficulty of balanced test construction. However, the faculty-authored cohort was three times more successful, yielding three (10.0%) ideal MCQs, whereas the LLM cohort collapsed to just one (3.3%) item. This further shows that LLMs struggle to balance cognitive complexity, clinical distractor plausibility, and student sorting capability simultaneously within a single assessment item.

This highlights the challenge of structural superficiality in AI-assisted assessment design. Because LLMs are trained predominantly on global, Western-centric medical datasets, their outputs often incorporate clinical terminologies or diagnostic assumptions that do not align with regional student proficiency or local epidemiology [15]. Faculty-authored items are naturally calibrated to the specific developmental stage of their student cohort and localized curricular expectations. Therefore, without faculty curation to inject regional clinical relevance, the resulting uncurated options often fail to engage localized student cohorts, leading to the high rate of NFDs and compressed discrimination observed in this study.

To contextualize these findings for dental educators, the performance of these items must be evaluated through Van der Vleuten’s utility framework [11], which stipulates that if any single parameter drops to zero or near-zero, the overall utility of the entire assessment program collapses. In this study, the faculty-authored items demonstrated superior internal consistency (*α* = 0.851) compared to LLM-generated items (*α* = 0.798). When combined with the significantly higher item discrimination capacity of the human cohort, faculty-authored examinations maintain the standard. Conversely, generating 30 items took the model less than an hour, whereas human authors required several days of cognitive effort. However, the concurrent analysis demonstrates that this significant gain in feasibility comes at a steep, unacceptable cost to psychometric validity. The multiplicative nature of Van der Vleuten's formula implies that deploying raw, uncurated LLM output yields a low overall utility due to the psychometric compromises.

Consequently, the practical implications of these findings point away from complete automation and strongly toward a hybrid faculty-AI collaborative framework. The fact that LLM-generated items were highly reliable, clinically accurate, and stylistically indistinguishable from faculty writing suggests immense utility in the preliminary phases of test construction. Writing high-quality MCQs is a notoriously time-consuming administrative task that is highly vulnerable to item-writer fatigue. Dental faculties can leverage optimized, single-prompt frameworks to instantly generate structured exam blueprints, comprehensive clinical case vignettes, and baseline options. The human academic can then step in as a vital curator, refining the distractors to inject clinical nuance and calibrating the overall item difficulty based on localized student performance data. This hybrid human-in-the-loop workflow protects the psychometric integrity of the examination while simultaneously reducing the administrative time burden on faculty.

While this study provides a controlled, single-blind comparison, certain limitations must be acknowledged. First, the evaluation was conducted using a convenience sample of dental interns at a single institution, which may restrict the immediate generalizability of the student performance metrics across different international curricula. Additionally, while adequate for preliminary item analysis, the sample size reflects a single cohort, and classical test theory parameters (such as item difficulty and discrimination) are sample-dependent and benefit from larger, multi-cohort sample sizes. Second, item generation was restricted to a single clinical specialty (endodontics), limiting direct transferability to non-clinical or basic science disciplines. Third, this evaluation was confined to the baseline, uncurated performance of a single frontier model (Claude Sonnet 5) using a standardized single-prompt framework; alternative open-source, commercial, or fine-tuned medical models, as well as multi-step prompting strategies, may yield different psychometric distributions. Nevertheless, the underlying psychometric principles, particularly the challenge of generating cognitively effective distractors, remain broadly relevant.

Finally, to ensure an unconfounded research design, no post-hoc expert curation of model outputs was permitted. While scientifically necessary for this baseline evaluation, this strict isolation does not reflect the optimized, hybrid human-in-the-loop workflows recommended for actual academic deployment. Future research should investigate whether advanced prompt engineering can mitigate the distractor deficit, as well as quantify the direct psychometric and temporal gains achieved when faculty curate LLM-drafted items compared to traditional manual drafting. Additionally, expanding to multi-center and multi-specialty evaluations across broader dental disciplines represents a critical direction to confirm the generalizability of these findings.

## Conclusions

While next-generation LLMs like Claude Sonnet 5 have achieved an extraordinary level of semantic sophistication, demonstrating high accuracy in linguistic formatting, clinical plausibility, and key answer selection, our empirical findings demonstrate that single-prompt, uncurated model outputs cannot yet replace the pedagogical depth of experienced academic faculty. Rather than being entirely flawed, the model functions effectively as a rapid drafting tool. However, the high prevalence of non-functioning distractors and the significantly compressed discrimination indices observed in the LLM-generated items demonstrate that structural authenticity does not equate to psychometric utility. Within the scope of this study, raw LLM outputs demonstrated 100% baseline clinical accuracy during pre-test auditing and exceptional stylistic authenticity (κ = 0.152). However, uncurated generation yielded only a 3.3% rate of psychometrically ideal items, compared to 10.0% for faculty items. The model is well suited to rapid preliminary drafting, but its high distractor failure rate (16.7% total collapse, where all distractors failed) and compressed discrimination indices (36.7% poor discriminators) make fully autonomous generation unviable for high-stakes assessments. The future of AI in health professions assessment lies not in autonomous generation, but in a collaborative, human-in-the-loop model. By pairing the rapid drafting capabilities of LLMs with the rigorous psychometric oversight, regional calibration, and clinical experience of academic educators, institutions can safely maximize efficiency without compromising the integrity of student assessments.

## Appendices

Appendix 1: prompt framework for generation of multiple-choice questions in endodontics using Claude Sonnet 5

You are an expert dental educator and endodontist. Please develop one single best-answer multiple-choice question (MCQ) to assess the endodontic knowledge of a dental intern student based strictly on the guidelines below.

Question Variety and Format Style

You have full autonomy to choose the question style and cognitive level. The question can range from a simple, direct, factual one-liner (testing "Remember/Understand") to a multi-sentence clinical vignette/patient case scenario (testing "Apply/Analyze/Evaluate"). Ensure the final question matches the complexity of the domain selected.

Stem and Lead-In Requirements

Contains only essential information required to reach the correct answer. No excessive or meaningless "red herrings." No abbreviations anywhere in the stem or options (e.g., write out "sodium hypochlorite" instead of "NaOCl"; write "periodontal ligament" instead of "PDL"). Must end with a clear, direct question testing a single clinical decision or concept.

Options (A-D): Exactly four options (one correct answer, three highly plausible distractors representing common student misconceptions or closely related clinical situations). All options must be homogeneous (same category, structural style, and approximate word length). No overlapping meanings; strictly avoid “all of the above” or “none of the above”. Randomize the position of the correct answer (do not default to A).

Quality Controls

Avoid grammatical clues or absolute terms (always, never). No negative phrasing (do not use "except" or "which of the following is not...") unless absolutely critical for clinical safety.

Output Format Parameters

Domain Selected, Question Style (State whether it is Factual/Conceptual or a Clinical Vignette/Scenario), Cognitive Level Targeted (State whether it is Remember, Understand, or Apply/Analyze/Evaluate), Question Stem, Lead-in Question, Option A, Option B, Option C, Option D, Correct Answer, Rationale for Correct Answer (Explain why it is the definitive best answer and briefly why the others are incorrect).

Generate the Question for This Target Endodontic Domain: (Insert Domain Here)

Appendix 2: the master endodontic domain list

Pulpal and Periapical Diagnosis

Patient history, clinical testing (thermal/electric pulp testing, percussion, palpation), and the establishment of a formal diagnosis (e.g., irreversible pulpitis, acute apical abscess) according to standardized classification systems.

Endodontic Radiography and Imaging

Interpretation of periapical radiographs, identifying anatomical landmarks, recognizing pathology, identifying internal/external resorption, and the clinical indications/limitations of Cone Beam Computed Tomography.

Case Assessment and Treatment Planning

Evaluation of case difficulty, assessing restorative restorability, tooth prognosis, management of medically compromised patients, and identifying when a case requires specialist referral.

Applied Clinical Anatomy and Morphologies

Internal tooth anatomy, common root canal configurations (e.g., Weine or Vertucci classifications), identifying accessory/lateral canals, location of the MB2 (second mesiobuccal) canal in maxillary molars, and variations in root shapes across different dentitions.

Access Cavity Preparation and Isolation

Proper outline forms for anterior and posterior teeth, selecting appropriate burs, preserving tooth structure, avoiding perforations, and mandatory rubber dam isolation principles.

Cleaning and Shaping (Chemo-Mechanical Debridement)

Kinematics and metallurgy of nickel-titanium (NiTi) rotary instruments, establishing working length, glide path creation, apical gauging, and instrumentation techniques (e.g., crown-down or step-back).

Endodontic Irrigation Protocols

Chemical properties, concentrations, and interactions of common irrigants (Sodium Hypochlorite, Ethylenediaminetetraacetic acid, Chlorhexidine), tissue dissolution capabilities, smear layer removal, and safe delivery/activation systems.

Obturation Materials and Techniques

Goals of root canal sealing, properties of gutta-percha, sealer categories (zinc oxide eugenol, resin-based, and bioceramics), and techniques like cold lateral compaction or warm vertical condensation.

Endodontic Mishaps and Complications

Management and prevention of clinical errors including instrument separation, root/furcation perforations, ledge formation, and sodium hypochlorite accidents.

Vital Pulp Therapy and Emergencies

Managing acute endodontic pain, emergency pulpotomy/pulpectomy, direct/indirect pulp capping, apexogenesis/apexification in immature teeth, and using bioceramic materials (Mineral Trioxide Aggregate/Biodentine).

Appendix 3: representative Claude Sonnet 5-generated MCQ example

Domain Selected: Pulpal and Periapical Diagnosis

Question Style: Clinical Vignette/Scenario

Cognitive Level Targeted: Apply/Analyze

Question stem: A 27-year-old man reports a two-day history of spontaneous, throbbing pain in his lower right first molar that intensifies when lying down at night. He also reports discomfort when chewing on that side. Clinical examination reveals a deep composite restoration on the tooth, with no swelling or sinus tract present. The tooth exhibits an intense, sharp response to a cold test that lingers for approximately forty-five seconds after the stimulus is removed. The tooth is mildly tender to percussion, and there is no mobility. A periapical radiograph shows an intact lamina dura and no periapical radiolucency.

Lead-in question: Based on these findings, which pulpal and periapical diagnosis best fits this presentation?

Option A: Pulp Necrosis with Symptomatic Apical Periodontitis Option B: Reversible Pulpitis with Normal Apical Tissues Option C: Symptomatic Irreversible Pulpitis with Normal Apical Tissues Option D: Symptomatic Irreversible Pulpitis with Chronic Apical Abscess

Correct answer: Option C (Symptomatic Irreversible Pulpitis with Normal Apical Tissues)

Rationale for correct answer: The prolonged, lingering pain to cold (well beyond the normal several-second response), spontaneous nocturnal pain, and history of deep restorative treatment are hallmark findings of symptomatic irreversible pulpitis, indicating the pulp is inflamed but still vital and responsive to stimuli. The mild percussion tenderness reflects early inflammatory spread into the periodontal ligament space, which can occur even with vital pulp tissue, but the intact lamina dura and absence of a periapical radiolucency confirm that the periapical tissues remain normal radiographically. Option A is incorrect because a positive, exaggerated response to cold confirms a vital, sensate pulp; necrotic pulps do not respond to thermal testing. Option B is incorrect because reversible pulpitis produces a brief, mild response to cold that resolves within a few seconds once the stimulus is removed, not a lingering forty-five-second response. Option D is incorrect because a chronic apical abscess implies a necrotic pulp with an associated periapical radiolucency or sinus tract, neither of which is present here, and the tooth would not respond to cold testing.
